# Protein Modularity of Alternatively Spliced Exons Is Associated with Tissue-Specific Regulation of Alternative Splicing

**DOI:** 10.1371/journal.pgen.0010034

**Published:** 2005-09-16

**Authors:** Yi Xing, Christopher J Lee

**Affiliations:** Molecular Biology Institute, and Institute for Genomics and Proteomics, Department of Chemistry and Biochemistry, University of California, Los Angeles, California, United States of America; North Carolina State University, United States of America

## Abstract

Recent comparative genomic analysis of alternative splicing has shown that protein modularity is an important criterion for functional alternative splicing events. Exons that are alternatively spliced in multiple organisms are much more likely to be an exact multiple of 3 nt in length, representing a class of “modular” exons that can be inserted or removed from the transcripts without affecting the rest of the protein. To understand the precise roles of these modular exons, in this paper we have analyzed microarray data for 3,126 alternatively spliced exons across ten mouse tissues generated by Pan and coworkers. We show that modular exons are strongly associated with tissue-specific regulation of alternative splicing. Exons that are alternatively spliced at uniformly high transcript inclusion levels or uniformly low levels show no preference for protein modularity. In contrast, alternatively spliced exons with dramatic changes of inclusion levels across mouse tissues (referred to as “tissue-switched” exons) are both strikingly biased to be modular and are strongly conserved between human and mouse. The analysis of different subsets of tissue-switched exons shows that the increased protein modularity cannot be explained by the overall exon inclusion level, but is specifically associated with tissue-switched alternative splicing.

## Synopsis

Alternative splicing is a biological process that generates multiple mRNA and protein variants through alternative combinations of protein-coding exons. It is a widespread mechanism of gene regulation in higher eukaryotes. In recent years, scientists have found that when an exon is observed to be alternatively spliced in multiple species, its length is much more likely to be an exact multiple of three nucleotides. Since each amino acid is encoded by three nucleotides, these exons can be inserted or removed from the transcript as a “modular” protein-coding unit, without affecting the downstream protein translation. However, the precise roles of these modular exons in gene regulation and genome evolution remain unclear.

Xing and Lee have now investigated these modular exons using high-throughput genomics data. They analyzed the mouse splicing microarray data from the research group of Dr. Benjamin Blencowe at University of Toronto. Exons whose alternative splicing levels vary dramatically across multiple tissues are much more likely to be modular exons and are highly conserved during human and mouse evolution. This study establishes a strong link between protein modularity of alternatively spliced exons and tissue-specific regulation of alternative splicing. It provides new insights into the function and regulation of alternative splicing and how it evolves.

## Introduction

Recently, there has been great interest in characterizing the functional selection pressures for alternative splicing by evolutionary genomics [1–4]. Ancestral alternative splicing events (i.e., alternative splicing events observed in multiple organisms) show evidence for strong functional constraints: these exons are more likely to be multiples of 3 nt in length [4–6], so the inclusion or exclusion of an exon does not disrupt the downstream protein reading frame or cause premature protein truncation; they are often flanked by highly conserved intronic sequences, suggesting increased selection for preserving important splicing regulatory signals [7,8]; the exon sequences are more conserved, indicated by increased nucleotide sequence identity [8–10]. These features have been explored extensively to discern functional alternative splicing events from splice variants generated by random spliceosomal errors and have been used successfully for predicting alternative splicing from raw genomic sequences [9,11,12].

In many of these studies, protein reading frame preservation has emerged as a valuable criterion for “functional” alternative splicing events [4,5,9,11], and it is interesting to ask what the significance of this “modular” class of exons is: that is, what general role they play in regulating the proteome and what distinguishes them from other alternative splicing events. Clearly, by maintaining the same protein reading frame regardless of whether the alternatively spliced exon is included or skipped, they enable a modular segment of amino acid sequence to be added or deleted from the protein product without altering the rest of the protein or inducing nonsense-mediated decay [13]. This modular pattern is not seen in constitutive exons and in alternatively spliced exons that are included in the majority of a gene's transcripts. Instead, it is strikingly associated with ancestral alternative splicing events (i.e., exons that are observed to be alternatively spliced in two or more species), most particularly those exons that are only included in a minority of a gene's transcripts [5]. However, the precise role of these modular alternative splicing events in genome evolution remains unclear.

To better characterize this interesting class of exons, we have analyzed microarray data for a large set of mouse genes containing 3,126 alternatively spliced exons, generated by Pan and coworkers [14]. Whereas previous analyses of modular exons used expressed sequence tag (EST) data to estimate each exon's “inclusion level” (fraction of the gene's transcripts that include that exon) as a single value summed over all tissue types (often based on only a small number of EST counts [3]), these microarray data permit accurate measurements of its varying inclusion levels in ten different tissue types [14]. This opens up the interesting question of tissue-specific regulation of alternative splicing [15,16].

Exons that are of particular interest in terms of tissue variation of alternative splicing are those that are strongly included in the transcripts in some tissues, but also are strongly excluded in some other tissues—suggesting that the alternative splicing of these exons is under significant change across different tissues and shows strong tissue specificity. In this paper, we refer to these exons as “tissue-switched” exons. We show that modular exons are strongly associated with tissue-switched alternative splicing. Comparing the exon sequences between human and mouse genomes, we demonstrate that many tissue-switched alternative exons are ancient and were under elevated selection pressure for both protein modularity (frame preservation) and sequence conservation during the recent mammalian evolution.

## Results

### Protein Reading Frame Preservation Is Associated with Tissue-Switched Exons

Using the mouse microarray data of Pan and colleagues [14], we analyzed the exon inclusion levels across ten tissues for 3,126 alternatively spliced exons in mouse. From the total set, 2,171 exons were assigned with a confident inclusion level in at least three tissues. We identified a total of 237 (11%) tissue-switched alternative exons according to our criteria (see Defining Categories of Tissue-Switched Exons from Microarray Data, in Materials and Methods), 605 exons that were major-form in all the tissues (always major), and 120 exons that were minor-form in all the tissues (always minor) (Table 1). These 962 exons were included in our further analyses. The size of our dataset is comparable to or substantially larger than a few other recent human–mouse comparative studies of alternative splicing [6,7,10,17,18], with information about these exons' splicing patterns at a much higher resolution (on average a confident inclusion level in seven tissues) compared to EST-based studies [3,5].

**Table 1:**
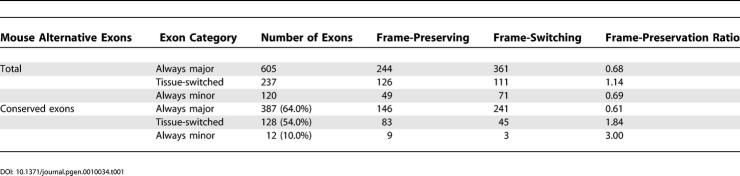
Frame Preservation Analysis for the Total and Conserved Sets of Always Major, Tissue-Switched, and Always Minor Exons

Examining the frame-preservation ratio for these exons, we observed that only tissue-switched exons had an overall association with protein frame preservation. Previous studies by Gilbert and colleagues [19,20] have shown that exons in the human genome are slightly more likely to be frame-preserving than expected by the random chance (0.5), yielding a background frame-preservation ratio of 0.64 (i.e., 39% of exons in the genome are frame-preserving [5]). The frame-preservation ratios measured for always major and always minor alternatively spliced exons were 0.68 and 0.69, respectively, almost the same as the background ratio observed for constitutive exons. By contrast, the frame-preservation ratio for tissue-switched exons nearly doubled (Table 1), a statistically significant result (*p* < 0.001 for tissue-switched exons versus always major exons; *p* = 0.017 for tissue-switched exons versus always minor exons; one-sided Fisher exact test).

### Tissue-Switched Exons Are Strongly Conserved

Since frame preservation has been observed to be associated primarily with ancestral alternative splicing events (i.e., alternative splicing events that have been observed in more than one species [5]), we tested the conservation of these exons between the mouse and human genomes (Table 1). Whereas always minor exons were conserved in only 10% of cases (in agreement with previous analyses [3,14]), tissue-switched exons showed a high rate of conservation (54%) similar to that of always major exons (64%). To control for the effect of exon length on our BLAST search, we restricted our analysis to a set of exons longer than 90 nt, and obtained similar results (data not shown).

To assess whether this pattern of conservation simply reflects the *overall* inclusion level of an exon (summed over all tissues), we further subdivided tissue-switched exons into three classes: usually major, observed to be the major-form in the majority of tissues; usually minor, observed to be a minor form in the majority of tissues; intermediate, observed to be neither the major-form in the majority of tissues, nor minor form in the majority of tissues. It should be emphasized that by definition all tissue-switched exons were major-form in at least one tissue *and* minor form in at least one other tissue. Among the 237 tissue-switched exons, we identified 40 usually major exons, and 37 usually minor exons by these criteria.

Analysis of the conservation of these exon types shows that tissue-switched alternative splicing, and not just a high overall inclusion level, is associated with a high rate of conservation (Figure 1A). Whereas always minor exons had a low rate of conservation (10%), usually minor exons had a high rate of conservation (62.2%) similar to that of always major exons (64%), despite the fact that they had a low overall inclusion level, only marginally higher than that of always minor exons. Overall, all types of tissue-switched exons had high rates of conservation similar to those of always major and constitutive exons.

**Figure 1:**
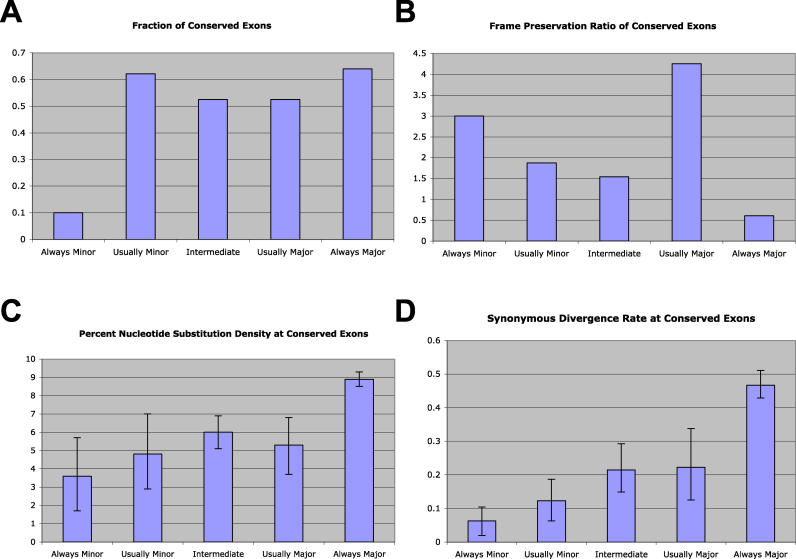
Increased Functional Constraints Are Associated with All Types of Tissue-Switched Exons (A) The fraction of exons conserved between human and mouse for each exon category. All types of tissue-switched exons (usually major, intermediate, usually minor) showed a high rate of conservation. (B) The frame-preservation ratio for each category of exons. Protein reading frame preservation is associated with all types of tissue-switched exons and conserved, always minor exons. Conserved, always major exons showed no evidence for increased protein reading frame preservation. (C) The percent nucleotide substitution density for each exon category. All types of tissue-switched exons showed a reduced nucleotide-sequence substitution density between human and mouse, similar to what is observed for conserved, always minor exons. Error bars represent 95% confidence intervals obtained from nonparametric bootstrapping. (D) The nucleotide substitution rate calculated for synonymous sites *(Ks)* from human and mouse orthologous exon sequences (see Materials and Methods). All types of tissue-switched exons showed reduced *K*s rates between human and mouse, similar to what is observed for conserved, always minor exons. Error bars represent 95% confidence intervals obtained from nonparametric bootstrapping.

### Protein Frame Preservation Is Strongly Associated with All Types of Conserved Tissue-Switched Exons

We evaluated the frame-preservation ratio for conserved tissue-switched exons, subdivided by different classes (Figure 1B). These data show that frame preservation is strongly associated with all the different types of tissue-switched exons. Intermediate and usually minor exons had a 3-fold higher frame-preservation ratio than always major exons (*p* < 0.001 for intermediate versus always major exons; *p* = 0.009 for usually minor versus always major exons; one-sided Fisher exact test). The largest increase was observed in usually major exons (a 7-fold increase; *p* < 0.001 for usually major versus always major exons), which differ in the microarray data only slightly from always major exons (indeed, observation of the exon to be minor-form in only a single tissue sample is sufficient to move it from the always major to the usually major category). These data suggest that frame preservation, like conservation, cannot be explained by the overall inclusion level, but instead is strongly associated with tissue-switched exons. Although 90% of the always minor exons were not conserved between human and mouse, those that were conserved also had a high frame-preservation ratio, consistent with previous studies using EST data [5].

Unusually high sequence identity in conserved exons has been observed to be another valuable indicator of functional alternative splicing [8–11], indicative of the presence of splicing regulatory elements within the exon sequence. We therefore examined the level of sequence identity within the different classes of conserved exons. Tissue-switched exons displayed a dramatic decrease in the density of nucleotide substitutions compared with always major exons, and similar to what is observed in a small number of conserved always minor exons (Figure 1C). To assess whether this difference might be attributable to amino acid-level selection pressure, we measured the nucleotide substitution density specifically at synonymous sites (where substitutions cause no change to the amino acid sequence). Tissue-switched exons displayed a greater than 2-fold decrease in substitution density (relative to always major exons), even at synonymous sites (Figure 1D). These data indicate that the protein-level functional selection pressure demonstrated by frame preservation is accompanied in tissue-switched exons by an additional selection effect that cannot be explained by amino acid selection, consistent with an increased abundance of splicing regulatory elements as previously demonstrated [8,21,22].

## Discussion

Frame-preserving alternative splicing events are of great functional interest because they produce a modular alteration of the protein product—adding or removing a single peptide segment without altering the rest of the protein sequence. Frame preservation has been proposed as evidence that an alternative splicing event is functional [4–6] and has proved valuable for predicting which exons in a genomic sequence are likely to be alternatively spliced [9,11,12]. Such alternative splicing events can have surprisingly sophisticated effects on protein structure, protein interactions, and function, as illustrated recently in the Piccolo C2A domain [23].

Our data suggest that this pattern of modular alternative splicing is strongly associated with tissue-switched exons. Analysis of microarray data from ten mouse tissues indicates that tissue-switched exons have the highest frame-preservation ratio, even for relatively subtle tissue-switching events. For example, whereas always major exons had a frame-preservation ratio near background (i.e., the ratio for constitutive exons in the mouse genome), exons that were usually major but observed to become the minor form in at least one tissue showed a 7-fold increase in frame preservation. Overall, the vast majority of nonrandom frame-preservation events (i.e., those above the number expected by chance) displayed tissue-switched alternative splicing even in the small panel of tissues (ten) analyzed here.

We have performed several control tests to evaluate the possibility of bias or artifacts due to the confidence-rank cutoff (recommended by Pan and colleagues) and classifiers (e.g., inclusion-level cutoffs for major versus minor form) applied to the dataset. Pan et al. recommended a cutoff of top-16,000 confidence ranks to identify confident exon inclusion levels [14]. To further exclude possible artifacts due to noise in the microarray experiment, we tested a more stringent filtering criterion (a cutoff of top-10,000 confidence ranks). These data robustly reproduced our original results. We also tested several different inclusion-level cutoffs for defining major versus minor forms (60% versus 40%, 66% versus 34%, and 75% versus 25%). These different cutoffs yielded consistent results. These control analyses demonstrate that our results are robust and are not artifacts of microarray noise or arbitrary cutoff values.

Tissue-switched exons combine several interesting features. On the one hand, they are strongly conserved, like constitutive and always major exons. Even usually minor tissue-switched exons showed a high frequency of conservation, similar to that of always major exons. On the other hand, tissue-switched exons display strong patterns of functional selection characteristic of ancestral minor-form alternative splicing, including strong frame preservation and reduced nucleotide substitution density. Even usually major exons had a frame-preservation ratio seven times that of always major exons, and a nucleotide substitution density 2-fold less than that of always major exons. This reduced level of substitution cannot be attributed to amino acid selection pressure, since it is also observed at synonymous codon positions. The fact that this pattern is observed specifically in tissue-switched exons suggests that it may reflect the presence of conserved regulatory motifs important for tissue-specific regulation of alternative splicing [24,25]. Increased sequence identity at alternatively spliced exons compared to constitutive exons has been reported as a predictive characteristic of alternative splicing [9,11,12] and has been shown to be associated with exonic splicing enhancer and splicing silencer sites [7,8,10,22]. This pattern of reduced nucleotide substitution appears to correlate quantitatively with the overall inclusion level for each exon (Figure 1C and 1D). Usually major exons had a synonymous substitution density of 0.223, intermediate exons 0.20, and always minor exons 0.06. This implies that restriction of an exon's expression to fewer and fewer tissues may require *more* regulatory sites.

These data also suggest several questions about ancestral alternative splice forms previously characterized by many groups [4–6,10,17,26]. Ancestral alternative splicing events (defined as alternative splicing observed in more than one species, and thus likely to be inherited from the common ancestor) show a similar profile of strong frame preservation [4,5], particularly for ancestral minor-form exons [5]. While the previous analysis pooled ESTs from all tissues to estimate the *overall* inclusion level of an alternative exon [5], in this study we used microarray data to obtain tissue-specific inclusion levels measured in ten different mouse tissues. These data show that it is not simply the overall inclusion level, but, more importantly, tissue-switched regulation of alternative splicing that is highly correlated with protein modularity. Therefore, our study reveals a strong link between protein modularity of alternative exons and tissue-specific regulation of alternative splicing. Consistent with previous studies, we observed that a small fraction of always minor mouse exons were conserved in the human genome and had a high frame-preservation ratio (3.0). One obvious question is whether many of these apparently always minor exons might actually be tissue-specific. Since only a small panel of ten mouse tissues was analyzed in the Pan et al. [14] microarray data, it is possible that some of these exons might be expressed as a major-form in other mouse tissues or individual cell types. A second possibility is that they are associated with transient regulatory events (i.e., a specific cellular activation state), rather than an individual tissue. Finally, the fact that these exons are conserved between human and mouse and have a high frame-preservation ratio similar to that previously reported for ancestral alternative splicing events [5] suggests that they may also be alternatively spliced in other species (such as human).

A minority of tissue-switched exons was not conserved between mouse and human, and these exons did not exhibit an elevated frame-preservation ratio. This raises several questions. What is the function of these mouse-specific tissue-switched exons, and why do they not show a bias for protein frame preservation as is seen in the conserved tissue-switched exons? One possibility is that the evolution of frame preservation for a given exon may be a slow process, so recently created, mouse-specific exons might not have had time to be converted in substantial numbers. Another possibility is that some of these exons may regulate function by inducing nonsense-mediated decay, as has been proposed by Brenner and colleagues [13,27].

## Materials and Methods

### Identification of alternative exons from mouse splicing microarray profile.

We identified tissue-switched alternative exons using data from a recent microarray analysis of alternative splicing in mouse [14]. Starting from 4,892 candidate alternative splicing events detected in ESTs, Pan and colleagues applied a set of filters to exclude errors and artifacts in the EST libraries. A total of 3,126 candidate alternative splicing events were included in their microarray design. Their dataset is thus a large and comprehensive collection of exon skipping events in the mouse genome, representing the vast majority of such events for which there was acceptable evidence. The exon inclusion level for these alternatively spliced exons was determined by microarray experiments across ten tissues [14]. Pan and colleagues assigned a confidence rank to each exon inclusion level, based on their statistical analyses of the splicing microarray data. According to their subsequent RT-PCR validation of the inclusion levels, they recommended a confidence rank of top 16,000 as a cutoff for confident exon inclusion levels. We followed this recommendation throughout this study, although our tests show that the use of a more stringent filter does not change our results significantly (see Discussion). We restricted our analysis to inclusion-level measurements within the top-16,000 confidence-rank cutoff and excluded exons with less than three tissue measurements meeting this criterion.

### Defining categories of tissue-switched exons from microarray data.

An exon was defined as the major-form in a tissue if its inclusion level was greater than 66% in that tissue, or as the minor form if its inclusion level was less than 34% [3]. Because we were interested in the variations of the exon inclusion levels across multiple tissues, we referred to an exon as always major if it was the major-form in every tissue (with a confident exon inclusion level). Similarly, we referred to an exon as always minor if it was a minor-form exon everywhere. We defined an exon to be a tissue-switched exon if its inclusion level was higher than 66% in some tissues and less than 34% in other tissues. For tissue-switched exons, we further defined an exon as usually major if it was a major-form exon in the majority of the tissues. Similarly, we defined an exon as usually minor if it was a minor-form exon in the majority of the tissues. Finally, we defined a tissue-switched exon as intermediate if it was neither usually major nor usually minor.

### Frame-preservation ratio analysis.

We defined an exon as frame-preserving if the length of the exon was a multiple of 3 nt, and as frame switching if not [5]. Inclusion or exclusion of a frame-preserving exon by alternative splicing leaves the downstream protein reading frame unchanged; for this reason, frame preservation has been proposed by several groups as evidence that an alternative splicing event is functional [5,6]. We calculated the frame-preservation ratio for a given set of exons as the number of frame-preserving exons divided by the number of frame-switching exons. We performed the Fisher exact test to assess whether the frame-preservation ratios for two groups of exons were significantly different.

### Comparative analysis of tissue-switched exons in human and mouse genomes.

To determine whether an exon was conserved between human and mouse, we searched the human genome using nucleotide BLAST [28]. We defined an exon as conserved in another genome if we obtained a significant hit (BLAST expectation value less than 10^−4^) from BLASTN, aligning to the full length of the mouse exon, with no more than 12 nt deletion. It should be emphasized that this differs somewhat from the criteria of Modrek and Lee, whose dataset was constrained to the subset of genes where the exons adjacent to the alternatively spliced exon were successfully mapped to the orthologous gene in humans [3]. Since the dataset presented here lacks that extra constraint, it gives somewhat lower conservation estimates than Modrek & Lee and similar conservation estimates to Pan and colleagues [14].

For alternative exons conserved across genomes, we calculated their percent nucleotide sequence identity between human and mouse. We also calculated their rates of synonymous divergence *(Ks),* following the protocol of Nekrutenko and colleagues [29]. Briefly, orthologous exon sequences from human and mouse were translated and then aligned using CLUSTALW under default parameters [30]. This protein alignment was used to seed an alignment of corresponding nucleotide sequences, and gaps in the alignment were trimmed. We estimated the *Ks* rate from the codon-based nucleotide sequence alignment using the yn00 program of the PAML package [31,32]. This method takes into account the transition/transversion bias and codon usage bias for estimating *Ks.* We performed the Wilcoxon rank sum test to assess whether the nucleotide sequence identity or *Ks* rate for different groups of exons showed a statistically significant difference.

## Abbreviations

EST: expressed sequence tag
*Ks*: rate of synonymous divergence
